# The Use of Metabolomes in Risk Stratification of Patients with Heart Failure: A Scoping Review

**DOI:** 10.3390/life16030514

**Published:** 2026-03-20

**Authors:** Umar G. Adamu, Marheb Badianyama, Minenhle Mayisela, Joel Amoni, Dineo Tsabedze, Muzi Maseko, Nqoba Tsabedze

**Affiliations:** 1 Division of Cardiology, Department of Internal Medicine, School of Clinical Medicine, Faculty of Health Sciences, University of the Witwatersrand, Johannesburg 2193, South Africa; marhebbadianyama@gmail.com (M.B.); minenhle.mayisela@wits.ac.za (M.M.); joel.amoni@wits.ac.za (J.A.); nqoba.tsabedze@wits.ac.za (N.T.); 2 Department of Nuclear Medicine, Sefako Mekgatho Health Sciences University, Pretoria 0204, South Africa; dineo.tsabedze@smu.ac.za; 3 Nutrition and Hypertension Laboratory, Department of Physiology, Faculty of Health Sciences, University of the Witwatersrand, Johannesburg 2193, South Africa; muzi.maseko@wits.ac.za

**Keywords:** metabolomes, metabolomics, heart failure phenotypes, risk stratification, mass spectrometry, nuclear magnetic resonance, personalized care

## Abstract

Heart failure (HF) is associated with substantial morbidity and mortality. Metabolic abnormalities are increasingly recognized as integral to HF pathophysiology and may provide incremental value for phenotyping and prediction of outcomes. However, a comprehensive synthesis of metabolic alterations and their prognostic implications remains limited. This scoping review aimed to map metabolic changes in HF, describe analytical methods, and evaluate their diagnostic and prognostic relevance for clinical risk assessment. Methods: We systematically searched PubMed, Scopus, Web of Science, Cochrane Central, and grey literature from January 2010 to December 2024 to identify studies evaluating metabolic profiling in patients with HF. Two independent reviewers screened studies using predefined inclusion criteria and data were extracted using a customized charting form. Discrepancies were resolved by consensus or a third reviewer. We reported and synthesized findings narratively in accordance with scoping review methodology. Results: Seventy-two studies (66 observational and 6 randomized) were included, encompassing HF phenotypes including HF with reduced ejection fraction (HFrEF), HF with mildly reduced ejection fraction (HFmrEF), and HF with preserved ejection fraction (HFpEF). The analytical approaches included mass spectrometry and nuclear magnetic resonance (^1^H-NMR) platforms. The main metabolite classes that demonstrated prognostic significance were amino acids, acylcarnitines, and lipids, and gut-derived metabolites, which were associated with mortality, HF hospitalization, or disease progression. Several studies reported incremental prognostic value beyond conventional biomarker; however, most were exploratory, with modest sample sizes, limited external validation, and heterogeneous methodologies. Conclusions: Metabolomic profiling identifies biologically relevant alterations predicted worse clinical outcomes in HF and may complement existing risk assessment strategies. Nevertheless, standardized workflows and large prospective validation studies are required before clinical implementation can be considered.

## 1. Introduction

Heart failure (HF) is a global health concern affecting 1–3% of the world’s population [1,2]. It is a heterogeneous clinical syndrome characterized by symptoms or signs from cardiac structural or functional abnormalities that impair the heart’s ability to pump blood to meet metabolic demands at normal filling pressure [3,4]. Despite advancements in understanding the pathophysiology of HF including earlier diagnosis and new therapies, HF remains a significant economic and societal burden, with persistently high morbidity and mortality [5]. These challenges underscore the need for continued research, especially to develop novel biomarkers to improve diagnosis, prognostication, and support personalized treatment strategies.

Currently, serum brain natriuretic peptide (BNP) and N-terminal pro-B-type natriuretic peptide (NT-proBNP) are the only biomarkers with a class 1A recommendation for routine use in the diagnosis HF [3,4]. However, their accuracy is affected by obesity, renal dysfunction, and age. Natriuretic peptides are more effective at excluding HF than confirming it and have limited use in guiding therapy [5,6,7,8,9,10]. Therefore, there is a pressing need to develop more cardiac-specific and reliable biomarkers that can improve diagnosis, risk stratification, and prognostication in HF.

Metabolomics enables comprehensive analysis of small-molecule metabolites derived from diverse metabolic pathways, including fatty acids, carbohydrates, amino acids, ketones, nucleosides, phospholipids, and polyamines across matrices such as plasma, urine, saliva, and tissue samples [11,12,13,14,15]. The identification of metabolomic signatures presents opportunities to discover new biomarkers, characterize HF phenotypes, assess disease severity, and elucidate mechanisms underlying HF progression. In acute decompensated heart failure, plasma metabolomic profiling demonstrated superior discrimination for rehospitalization and heart failure-related events compared with BNP alone. A composite metabolite panel comprising tetradecenoylcarnitine, dimethylxanthine, phenylacetylglutamine, and hypoxanthine achieved significantly higher predictive performance (AUC 0.87 vs. 0.60; *p* < 0.01), supporting potential incremental value in prognostic modelling [16].

In this review, risk stratification refers to the prediction of clinically relevant adverse outcomes, including all-cause mortality, cardiovascular mortality, heart failure hospitalization, rehospitalization, and composite endpoints incorporating these events. Phenotypic classification based on metabolomic profiles is discussed separately as a diagnostic or biological application.

Accordingly, this scoping review aims to map metabolomic alterations in heart failure with specific emphasis on their diagnostic utility and prognostic value for risk stratification, while providing necessary mechanistic context.

## 2. Materials and Methods

The scoping review used Arksey and O’Malley’s framework and adhered to PRISMA-ScR and the updated Joanna Briggs Institute (JBI) Methods Manual [17,18]. The protocol for this review was prospectively registered with the Open Science Framework (OSF; https://osf.io/sp6xj, accessed on 1 August 2023) and published a priori [19]. The protocol outlined the research questions, eligibility criteria, search strategy, data extraction procedures, and charting methods. Minor deviations from the original protocol were made. Although the planned search period was from 2010 to September 2023, it was extended to December 2024 to capture recent developments in metabolomics. These modifications did not alter the review’s objectives, research questions, or overall findings.

### 2.1. Eligibility Criteria

We used the Population, Concept, Context (PCC) framework to set our inclusion criteria, following Joanna Briggs Institute guidance [19].

#### 2.1.1. Participants

We included studies involving adults (≥18 years) with HF from any cause, including both ischemic and non-ischemic origins. Studies were excluded if they had participants under 18 years, used animal models, or tested samples other than blood.

#### 2.1.2. Concept

The focus was on metabolomics in HF management, including diagnosis, prognosis, and follow-up. The review examined metabolic alterations in the systemic circulation of patients with HF and, where applicable, metabolic interactions with either the myocardium or the intestine. We also documented the analytical platforms and methods used to measure metabolites. The review aimed to map these approaches without formally appraising or critiquing the analytical methodologies.

#### 2.1.3. Context

We included studies from inpatient, outpatient, and community settings worldwide. Only English-language studies were eligible.

### 2.2. Types of Sources

The eligible study designs included randomized controlled trials, non-randomized interventional studies, observational cohort studies, cross-sectional studies, descriptive studies, and relevant scoping or systematic reviews. We excluded case reports, case series, animal studies, studies with fewer than 10 participants or that did not involve humans, non-English publications, study protocols, and studies without full text. Only full-text articles published from January 2010 to December 2024 were included. We chose January 2010 as the start date because metabolomic literature became much more robust, and older papers may no longer be relevant.

### 2.3. Information Sources and Search Strategies

The literature search was conducted in consultation with a medical librarian, initially in December 2022, and the final search was done in December 2024. We initially searched PubMed following the Joanna Briggs Institute’s recommended three-step method [20]. A PubMed search was conducted using index terms and text words derived from the titles and abstracts of the relevant studies. The key search terms included “heart failure,” “myocardial failure,” “metabolomes,” “metabonomic*,” “analytical technique,” and related synonyms. These keywords and index terms were subsequently combined using appropriate Boolean operators (‘AND’, ‘OR’, ‘NOT’) and Medical Subject Headings for comprehensive searching across four major databases: PubMed, Scopus, Web of Science, and Cochrane Central. The detailed search strategy used for all the databases is shown in Table S1. To supplement the electronic search, three grey literature sources, Google Scholar, the WHO Library, and OpenGrey were also queried. The search strategy was independently verified by two reviewers (U.G.A. and M.B.). Additional relevant studies were identified through backward snowballing of the reference lists of the retrieved articles. We did not contact the authors for the full text.

### 2.4. Study Selection

All identified citations were imported into the Rayyan software (version 1.7.2) and the duplicate records were removed prior to screening. Two reviewers (U.G.A and M.B.) independently screened titles and abstracts against the review questions and eligibility criteria. The full texts of potentially eligible studies were subsequently retrieved and assessed for inclusion. Any disagreements were resolved by discussion and consensus, with arbitration by a third reviewer if required (J.A., D.T., M.M., or N.T.).

### 2.5. Data Extraction, Analysis, and Presentation

Three reviewers (U.G.A., M.B., and M.M.) independently extracted data from the included studies using a standardized data extraction form developed by the review team. The form was pilot tested and refined iteratively during the extraction stage. Revisions to the form were documented, and any additional categories that emerged during extraction were discussed and incorporated into the charting process. Data extraction captured population characteristics (first author, year of publication, study design, and country) and key conceptual elements, including sample size, biological sample source, HF phenotypic distribution, analytical methods, metabolomic domains assessed, and outcomes. Disagreements during data extraction were resolved by consensus or consultation with a third reviewer (T.D., M.M., or N.T.). In line with scoping review methodology, this review focused on mapping and thematically summarizing the available evidence rather than assessing study quality, risk of bias, or intervention effects.

## 3. Results

### 3.1. Study Inclusion

**Figure 1:**
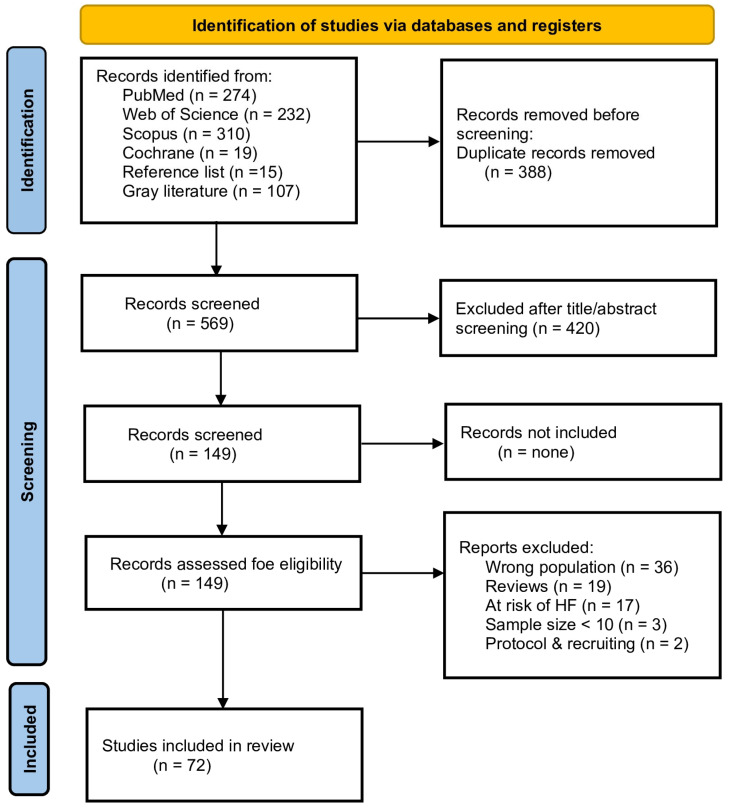
PRISMA-ScR flow diagram of study screening and selection.

The study selection process is illustrated in the PRISMA-ScR flow diagram (Figure 1). The database search identified 957 records, including those from PubMed (n = 274), Scopus (n = 310), Web of Science (n = 232), and Cochrane Central (n = 19). Additional records were identified through hand searches of reference lists (n = 15) and grey literature (n = 107). After removal of 388 duplicates, 569 records remained for title and abstract screening, of which 420 were excluded. The full texts of 149 articles were assessed for eligibility, and 72 studies met the inclusion criteria and were included in the final review.

### 3.2. Characteristics of the Included Studies

Sixty-six (91.7%) of the included studies employed observational designs [16,21,22,23,24,25,26,27,28,29,30,31,32,33,34,35,36,37,38,39,40,41,42,43,44,45,46,47,48,49,50,51,52,53,54,55,56,57,58,59,60,61,62,63,64,65,66,67,68,69,70,71,72,73,74,75,76,77,78,79,80,81,82,83,84,85], while 8.3% were randomized controlled trials [86,87,88,89,90,91]. Approximately one-third of the studies (39%) were published between 2023 and 2024. Studies were conducted predominantly in Asia (n = 32, 44.4%), followed by the Americas (n = 20, 27.8%), and Europe plus the United Kingdom (n = 20, 27.8%). At the country level, China contributed the largest number of studies (n = 21, 29.2%), followed by the United States (n = 16, 22.2%). Additional contributions were from Taiwan (n = 6, 8.3%) and Germany (n = 6, 8.3%), followed by Italy (n = 4, 5.6%), Japan (n = 3, 4.2%), and Canada (n = 3, 4.2%). Russia and Poland each contributed 2 studies (2.8%), while Australia, Belgium, Brazil, Croatia, France, the Netherlands, New Zealand, and Spain each contributed 1 study (1.4%). The study populations included patients with acute and chronic HF, with several studies enrolling mixed cohorts and others focusing on specific phenotypes defined by left ventricular ejection fraction. The sample sizes varied widely across studies, consistent with the exploratory nature of metabolomics research in HF (Table S2).

### 3.3. Cardiac Energy Metabolism in Health and Disease

The myocardium is a highly energy-dependent organ that requires a continuous supply of adenosine triphosphate (ATP) to sustain contractile function. Its entire ATP pool is turned over approximately every 10–15 s. Nearly 95% of myocardial ATP is generated through mitochondrial oxidative phosphorylation, with fatty acids contributing approximately 40–70% [92,93,94,95,96,97]. Additional substrates, including lactate, ketone bodies, and amino acids, provide the remaining oxidative energy requirements, and approximately 5% of ATP is derived from anaerobic glycolysis [98,99,100]. A defining feature of the normal myocardium is metabolic flexibility, the ability to switch between energy substrates in response to changes in workload, nutrient availability, and hormonal signalling [3,100,101].

In case of myocardial injury, the heart undergoes structural and metabolic remodelling, a central pathological process in the development of HF [102]. Subsequently, this triggers various reactions that are characterized by impaired myocardial substrate utilization, mitochondrial dysfunction, and loss of metabolic flexibility [21,103]. These disturbances lead to the accumulation of metabolic intermediates, increased oxidative stress, and impaired coupling between substrate oxidation and ATP synthesis, and ultimately the development of an “energy-starved heart” [3,9,92]. In diabetes mellitus, insulin resistance impairs myocardial glucose uptake and oxidation, increasing reliance on fatty acid oxidation and further worsening metabolic flexibility [104].

### 3.4. Analytical Platforms for Metabolomic Profiling in Heart Failure

Metabolomic profiling employs high-resolution analytical platforms capable of detecting and quantifying small-molecule metabolites (typically <1500 Da) in biological matrices, including blood and its derivatives, urine, feces, and myocardial tissues [105]. The most widely used techniques are mass spectrometry (MS)-based approaches and nuclear magnetic resonance (^1^H-NMR) spectroscopy, each with distinct strengths and limitations [106,107] (Table 1).

Mass spectrometry is commonly coupled with chromatographic separation methods, such as liquid chromatography (LC-MS) or gas chromatography (GC-MS), to enhance metabolite separation, sensitivity, and coverage [105,106]. In contrast, ^1^H-NMR spectroscopy provides a complementary approach characterized by minimal sample preparation, high analytical reproducibility, and non-destructive analysis, albeit with lower sensitivity than MS-based methods [107,108]. Both MS- and ^1^H-NMR-based platforms can be applied using either targeted or untargeted analytical strategies, depending on the study objectives [109,110].

Recently, integrative multi-omics approaches, particularly the combined analysis of metabolomic and proteomic data, have been applied in HF populations, including HFpEF, to elucidate metabolic, inflammatory, and immune-related pathways underlying disease heterogeneity and progression [111].

### 3.5. Alterations in Energy Metabolism and Substrate Utilization

Across multiple studies, HF was reported to be consistently characterized by disturbances in myocardial energy metabolism, including impaired fatty acid oxidation, altered tricarboxylic acid (TCA) cycle intermediates, and increased reliance on alternative fuels such as ketone bodies [31,36,55,57,60,76,77,79,86]. The accumulation of short-, medium-, and long-chain acylcarnitines was a frequent finding in patients with HF, reflecting mitochondrial dysfunction and incomplete β-oxidation [22,34,42,45,52,57,64,77,86,88]. Elevations in ketone bodies and lactate are associated with disease severity and increased mortality in both reduced- and preserved-ejection fraction phenotypes [24,32,57,82,84,86] (Table 2).

### 3.6. Amino Acid Metabolism

Alterations in amino acid metabolism were among the most reproducible findings across the included studies. Dysregulation of branched-chain amino acids, aromatic amino acids, and urea cycle intermediates was observed in both acute and chronic HF [22,27,30,37,42,44,53,86]. Reduced circulating valine and histidine levels were independently associated with increased short- and long-term mortality [35,63,73], whereas elevated kynurenine and phenylalanine levels reflect heightened inflammatory and catabolic states [27,48,79].

### 3.7. Lipidomics and Acylcarnitine Profiles

Lipidomic analyses demonstrated widespread perturbations in phospholipids, sphingolipids, cholesterol derivatives, and lipoprotein particles [27,30,34,37,42,45,64,67]. Long-chain acylcarnitines were repeatedly associated with myocardial remodelling, exercise intolerance, and poor prognosis [27,42,57,85]. Altered high-density lipoprotein particle size and composition were linked to cardiovascular mortality, suggesting metabolic vulnerability beyond traditional lipid measurements [66,67].

### 3.8. Inflammation, Oxidative Stress, and Polyamine Pathways

Several metabolites implicated in inflammation and oxidative stress were associated with disease severity and prognosis. Arachidonic acid-related metabolites demonstrated both prognostic and potential therapeutic relevance [21,28]. Polyamine metabolism, including elevated N8-acetylspermidine, was found to predict worse clinical outcomes in ischemic cardiomyopathy [69]. Markers of oxidative lipid modification and redox imbalance were also observed in erythrocytes and plasma of patients with HF [87].

### 3.9. Gut Microbiome-Derived Metabolites

Multiple studies highlighted the role of gut-derived metabolites in the pathophysiology of HF. Elevated levels of trimethylamine-N-oxide, indoxyl sulfate, and p-cresyl sulfate were associated with disease severity and mortality [31,34,83]. Integrative microbiome-metabolome analyses demonstrated distinct metabolic signatures in ischemic versus non-ischemic cardiomyopathy [21,33].

### 3.10. Phenotyping and Risk Stratification

Metabolomic signatures distinguished HF phenotypes across the ejection fraction spectrum, with HF with mildly reduced ejection fraction (HFmrEF) and HF with preserved ejection fraction (HFpEF) demonstrating distinct metabolic profiles compared with HF with reduced ejection fraction (HFrEF) [30,37,45,64,78]. Several studies showed that metabolomic panels and composite risk scores provided incremental prognostic value beyond natriuretic peptides and conventional clinical variables [24,66,73,79,89]. A consolidated overview of the major metabolite classes, direction of change, associated clinical outcomes, predominant HF phenotypes, and analytical platforms is presented in Table 3.

### 3.11. Therapeutic and Mechanistic Insights

Targeted metabolomic profiling revealed treatment-associated metabolic shifts, particularly with sodium-glucose cotransporter-2 inhibitors and mechanical circulatory support [49,51,58,60,62,68,75,76,87]. However, the findings were heterogeneous, and some interventions, such as cardiac resynchronization therapy, did not demonstrate consistently metabolomic as predictors of response [80].

## 4. Discussion

**Figure 2:**
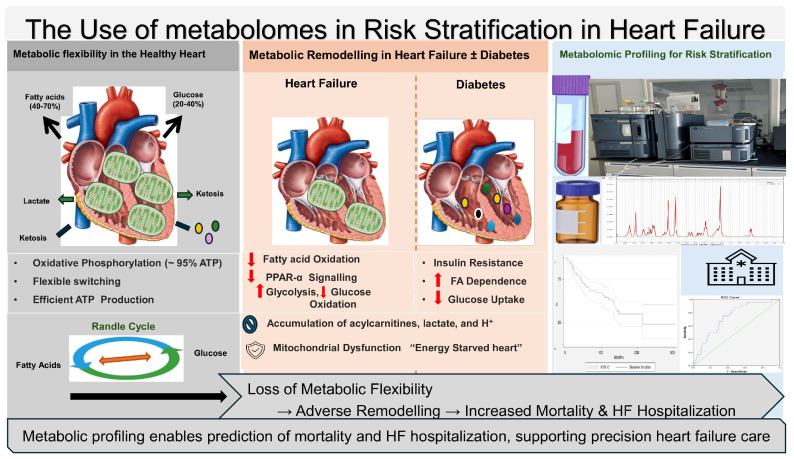
The use of metabolomes in risk stratification in heart failure.

In this scoping review, we synthesized evidence on metabolomic alterations in HF, interpreting findings related to analytical platforms and across three interrelated domains: mechanistic pathophysiology, diagnostic discrimination, and prognostic risk stratification, defined as prediction of mortality and heart failure hospitalization outcomes. Most of the included studies were exploratory, with limited external validation and a relative underrepresentation of low- and middle-income populations. Nevertheless, across diverse cohorts and study designs, metabolomic profiling consistently captures disease-relevant biological processes that are not fully reflected by conventional biomarkers or imaging modalities. Alterations in myocardial energy metabolism emerged as a dominant and highly consistent finding in the included studies. Analytical approaches vary widely, including LC-MS, GC-MS, and ^1^H-NMR spectroscopy (Figure 2).

From a mechanistic perspective, disturbances in myocardial energy metabolism were the most consistent findings. The impairment in fatty acid oxidation, altered TCA cycle intermediates, and increased reliance on alternative substrates, particularly ketone bodies, were reported across acute and chronic HF cohorts [31,37,41,57,84,86]. Elevated circulating acylcarnitines are frequently associated with adverse clinical outcomes and likely reflect impaired mitochondrial β-oxidation and loss of metabolic flexibility, which appear to be a central feature of HF pathophysiology [34,42,46,65,85,86]. Importantly, these metabolic disturbances provide mechanistic insights that extend beyond traditional left ventricular ejection fraction-based classification. Furthermore, perturbations in amino acid metabolism, including branched-chain amino acids, aromatic amino acids, and urea cycle-related metabolites, were consistently reported and associated with poor outcomes [27,30,73,74,79,86]. Reduced circulating levels of valine, histidine, and related metabolites are linked to disease severity and mortality, likely reflecting heightened catabolic stress, systemic inflammation, and impaired mitochondrial function [35,61,63,73,79].

In the healthy myocardium, metabolic flexibility allows dynamic substrate switching to maintain efficient ATP production. In heart failure (with or without diabetes), mitochondrial dysfunction and altered substrate utilization can lead to loss of metabolic flexibility and adverse remodeling. Metabolomic profiling using mass spectrometry and ^1^H-NMR platforms detects pathway-level disturbances, including alterations in acylcarnitines, ketone bodies, amino acids, and lipid species. These metabolite signatures provide biologically informed phenotyping and support prognostic risk stratification, including prediction of mortality and heart failure hospitalization, thereby contributing to precision heart failure management.

In addition, lipidomic profiling revealed widespread disturbances in phospholipids, sphingolipids, and cholesterol-related metabolites [45,52,76,77,79]. Long-chain acylcarnitines and altered lipid species are associated with myocardial remodelling, reduced functional capacity, and adverse prognosis [24,42,60,65,85]. Several studies demonstrated incremental prognostic value when lipidomic markers were incorporated into established clinical risk models, supporting lipid metabolism as a biologically informative axis for risk stratification in HF [24,30,89].

The metabolites related to inflammatory and oxidative stress pathways were also commonly reported [28,69,87]. Arachidonic acid derivatives, oxidized lipid species, and polyamines are associated with disease severity and mortality, underscoring the capacity of metabolomics to capture inflammatory and redox processes not assessed by traditional cardiac biomarkers [31,69]. Furthermore, gut microbiota-derived metabolites, including trimethylamine-N-oxide, indoxyl sulfate, and p-cresyl sulfate, were investigated in multiple studies [83]. These metabolites were linked with increased HF severity, renal dysfunction, and higher risk of adverse events, supporting the concept of a heart-gut-kidney metabolic axis [72]. Together, these findings reinforce metabolomics as a toll for pathway-level characterization of HF biology.

Furthermore, several studies also explored the diagnostic potential of metabolomic signatures. The metabolite panels revealed both common and phenotype-specific alterations across the ejection fraction spectrum [30,37,45,64,78]. In HFrEF, elevations in LCACs, ketone bodies, BCAAs, and TCA intermediates indicate impaired fatty acid oxidation and mitochondrial dysfunction [32,42,48,57,60,65,77,86,88]. In HFpEF, lipid remodelling, HDL alterations, myoinositol, kynurenine, and gut-derived metabolites were more prominent, consistent with inflammatory and metabolic-vascular dysregulation [21,32,37,41,53,72,83,86]. HFmrEF exhibits intermediate amino acid and acylcarnitine patterns, supporting biological overlap between HFrEF and HFpEF phenotypes [30,64].

Most importantly from a clinical perspective, multiple investigations evaluated the prognostic and risk stratification value of metabolomic markers. Associations between metabolite signatures and mortality or rehospitalization were consistently reported [24,66,73,79,89]. Incorporation of lipidomic and amino acid markers into established clinical models improved discrimination beyond natriuretic peptides and conventional variables [24,30,89]. Across phenotypes, recurrent disturbances in acylcarnitines, valine, phenylalanine, lactate, and polyamines suggest shared pathways of mitochondrial stress and substrate dysregulation that may provide incremental prognostic information [24,35,66,73,79]. These data support the potential role of metabolomics in refined risk stratification and biologically informed phenotyping in management strategies.

Since this review followed a scoping methodology, our objective was to map how metabolomic studies in HF were conducted rather than to formally appraise methodological quality. Across the included studies, adjustment for key metabolic confounders, such as renal function, diabetes, and background pharmacotherapy, was variably reported. Several studies described multivariable modelling incorporating clinical covariates [24,31,66,72,73,79,83,89], whereas others did not clearly specify adjustment strategies. The reporting of contemporary HF therapies, including SGLT2 inhibitors and mechanical support, was also heterogeneous [49,57,62,86]. This variability underscores the need for standardized reporting and prospective validation to enhance the clinical interpretability and translational relevance of metabolomic signatures in HF.

This scoping review has several limitations. First, consistent with scoping methodology, we mapped the available evidence without formally assessing study quality or measuring effect sizes. Consequently, no formal risk-of-bias assessment or meta-analysis was conducted, and the strength of individual associations was not determined. Second, there was significant heterogeneity among the included studies with respect to patient populations, HF phenotypes, disease severity, and clinical settings. Differences in analytical platforms, such as targeted versus untargeted metabolomics, and variations in sample preparation, metabolite coverage, and data normalization further limit direct comparability and reproducibility of studies. Third, most of the included studies were observational and exploratory, frequently single centre, with modest sample sizes and limited external validation. These characteristics limit causal inference and may increase the susceptibility to overfitting, particularly in multivariable or machine learning based metabolomic models. Consequently, reported associations may reflect population-specific patterns that are not broadly generalizable. In addition, the predominance of studies from high-income regions restricts global applicability. This limited geographic representation may have important implications for external validity. Metabolic phenotypes are influenced by ethnicity, dietary patterns, environmental exposures, socioeconomic factors, and comorbidity profiles such as diabetes and chronic kidney disease, which differ substantially across populations. Consequently, metabolomic signatures identified predominantly in high-income cohorts may not fully capture the biological heterogeneity of HF in underrepresented regions. Broader inclusion of diverse populations is therefore necessary to ensure generalizability and to avoid population-specific risk models that may not perform consistently across settings.

Fourth, inconsistent reporting of metabolite thresholds, reference ranges, and statistical modelling approaches has hindered the development of standardized and clinical applications. Although several studies have reported incremental prognostic value beyond that of conventional biomarkers, few have assessed their clinical utility in prospective or interventional settings.

Finally, publication bias cannot be ruled out, as studies reporting significant metabolomic associations are more likely to be published than those that do not. Despite comprehensive database and grey literature searches, relevant unpublished or negative studies may have been missed.

## 5. Conclusions

This scoping review demonstrates that metabolomic profiling consistently identifies alterations in amino acid, lipid, acylcarnitine, ketone body, and gut microbiota-related pathways across HF phenotypes. These findings provide mechanistic insight, support biologically informed phenotyping, and suggest potential incremental value for prognostic risk stratification beyond traditional clinical and biomarker-based approaches.

However, heterogeneity in study design, analytical platforms, and reporting standards, together with limited external validation and modest sample sizes, currently restrict clinical implementation. Standardized methodologies and large, prospective validation studies are required before metabolomics can be integrated into routine HF care.

## 6. Implications and Future Directions

Metabolomics offers a promising framework for precision HF management by enabling pathway-level characterization and potentially improving outcome prediction. Recurrent alterations in amino acids, acylcarnitines, and lipid metabolites suggest biologically coherent targets for refined risk stratification.

Future research should prioritize multicenter prospective validation, development of standardized analytical workflows, and establishment of clinically actionable thresholds. Integration with multimodal data, such as genomics, proteomics, and imaging may further enhance predictive performance and clarify disease mechanisms. Ultimately, interventional studies are required to determine whether metabolomics-guided strategies improve clinical outcomes and inform therapeutic decision-making.

From a translational perspective, practical considerations such as cost, turnaround time, and feasibility must also be addressed before integrating it. Most current metabolomic analyses rely on advanced platforms such as LC-MS or ^1^H-NMR, which require specialized infrastructure and trained personnel. These requirements may limit accessibility, particularly in resource-constrained settings, and may result in longer turnaround times compared with conventional biomarkers. Development of simplified targeted metabolite panels and integration into existing clinical laboratory workflows may improve feasibility for implementation in routine HF clinics.

## Figures and Tables

**Table 1:** Comparison of Analytical Platforms for Metabolomics in Heart Failure.

Feature	Mass Spectrometry	Nuclear Magnetic Resonance
Sample preparation	Extensive; often requires extraction, derivatization	Minimal; non-destructive
Metabolic coverage	Broad; detects lipids, acylcarnitines, amino acids, and small polar metabolites	Moderate; optimal for abundant and aqueous metabolites/compounds
Typical use in HF studies	Targeted or untargeted profiling for biomarker identification	Verification, longitudinal monitoring, and metabolite quantification
Quantification	Primarily relative and absolute quantification requires standards	Absolute quantification is straight forward
Suitability for risk stratification	High sensitivity enables detection of low abundance prognostic metabolites	Suitable for longitudinal monitoring and reproducible quantification
Data complexity	High; may require advanced bioinformatics for metabolite identification	Moderate; spectra generally easier to interpret
Reproducibility	Moderate; influenced by ionization and matrix effects	High; robust across runs and longitudinal studies
Sensitivity	High; can detect low abundance metabolites	Lower sensitivity, limited detection of subtle changes
Limitations	Platform-specific variability, ion suppression, complex data analysis	limited sensitivity and metabolites coverage

Abbreviations: HF = heart failure. Legend: Comparison of major analytical platforms used in metabolomic studies of heart failure, highlighting differences in metabolite coverage, sensitivity, reproducibility, and suitability for biomarker discovery and risk stratification.

**Table 2:** Phenotype-specific Metabolic Alterations Across the Ejection Fraction Spectrum in Heart Failure.

HF Phenotype	Reported Metabolites	Pathways	Clinicopathophysiological Interpretation	Key References
HFrEF	LCACs, SCACs, ketone bodies, BCAAs, 2-oxoglutarate	Impaired FAO; mitochondrial dysfunction; altered TCA cycle; enhanced ketone utilization	“Energy-starved” phenotype with reduced oxidative capacity and accumulation of incomplete β-oxidation products	[24,42,48,57,60,65,77,86,88]
HFmrEF	Amino acid perturbations, mixed ACNs and lipid profiles	Intermediate metabolic remodelling, partial mitochondrial impairment	Metabolic profile overlapping with HFrEF and HFpEF	[30,64]
HFpEF	Lipid species (phospholipids, HDL alterations), myo-inositol, kynurenine, gut-derived metabolites (TMAO, indoxyl sulfate), oxidized lipid	Lipid remodelling; inflammation, oxidative stress, endothelial and microvascular dysfunction, gut–heart metabolic axis	Systemic inflammatory-metabolic phenotype with preserved EF but altered substrate handling	[21,31,37,41,53,72]
Shared across phenotype	ACNs, AAs (valine, phenylalanine), lactate, polyamines	Mitochondrial stress, altered substrate utilization, catabolic activation, redox imbalance	Core metabolic remodelling common across HF irrespective of EF classification	[24,35,66,73,79]

Abbreviations: AAs = amino acids, ACNs = acylcarnitines, BCAAs = branched-chain amino acids, EF = ejection fraction, FAO = fatty acid oxidation, HDL=high density lipoprotein cholesterol, HF = heart failure, HFmrEF = heart failure with mildly reduced ejection fraction, HFpEF = heart failure with preserved ejection fraction, HFrEF = heart failure with reduced ejection fraction, LCACs = long-chain acylcarnitines, SCACs = short-chain acylcarntines, TMAO = trimethylamine n-oxide Legend: This table summarizes metabolite classes and dominant metabolic pathways reported across heart failure phenotypes defined by left ventricular ejection fraction. HFrEF is characterized by mitochondrial dysfunction and impaired fatty acid oxidation, whereas HFpEF demonstrates greater lipid remodelling and inflammatory-metabolic perturbations. HFmrEF exhibits intermediate and overlapping metabolic features. These distinctions support the potential role of metabolomics in precision phenotyping and risk stratification.

**Table 3:** Summary of Metabolomic Alterations Associated with HF Outcomes.

Metabolic Class	Direction	Outcome Association	Predominant Phenotype	Analytical Platform
Acylcarnitines	↑	Mortality, HF hospitalization	HFrEF > HFmrEF	LC-MS
Ketone bodies	↑ utilization	Disease severity, clinical outcomes	HFrEF	LC-MS, ^1^H-NMR
BCAAs	↑/↓	Mortality, functional decline	HFrEF, HFmrEF	LC-MS
Aromatic amino acids	↑	Mortality	All phenotypes	LC-MS
Lipid species	Altered	Remodelling, adverse prognosis	HFpEF, HFrEF	LC-MS
HDL-related metabolites	Altered	Cardiovascular mortality	HFpEF	^1^H-NMR
Lactate	↑	Acute HF severity	HFrEF	^1^H-NMR
Gut-derived metabolites	↑	Mortality, renal dysfunction	HFpEF	LC-MS

Abbreviations: BCAAs = branched-chain amino acids, ^1^H-NMR = nuclear magnetic resonance spectroscopy, HDL = high density lipoprotein cholesterol, HF = heart failure, HFmrEF = heart failure with mildly reduced ejection fraction, HFpEF = heart failure with preserved ejection fraction, HFrEF = heart failure with reduced ejection fraction, LC-MS = liquid chromatography mass spectroscopy. Legend: Summary of recurrent metabolomic alterations reported in heart failure, including direction of change, associated clinical outcomes (e.g., mortality and HF hospitalization), predominant HF phenotypes, and analytical platforms used for metabolite detection.

## Data Availability

The original contributions presented in the study are included in the article/Supplementary Material; further inquiries can be directed to the corresponding author.

## Supplementary Materials

The following supporting information can be downloaded at: https://www.mdpi.com/article/10.3390/life16030514/s1, Table S1: Search strategy for all databases; Table S2: Characteristics of included studies.

## Abbreviations

ATP	Adenosine triphosphate
HF	Heart failure
^1^H-NMR	Nuclear magnetic resonance spectroscopy
JBI	Joanna Briggs Institute
LC-MS	Liquid chromatography mass spectrometry
NT-proBNP	N-terminal pro-B-type natriuretic peptide
TCA	Tricarboxylic acid
