# Supplementary material for: The Use of Metabolomes in Risk Stratification of Patients with Heart Failure: A Scoping Review
Source: Life (Basel). 2026 Mar 20;16(3):514. doi: 10.3390/life16030514 (PMC13027496; doi:10.3390/life16030514)
Supplement: Supplementary file 1 [file life-16-00514-s001.zip › life-4175827-supplementary.pdf]

Supplementary Material

Table S1 **Search strategy for all databases**

Table S2 **Characteristics of included studies**

Table S1. Search strategy for all databases

Database	Search terms	Search results
PubMed	("Heart Failure"[Mesh] OR "heart failure" OR "cardiac failure" OR "myocardial failure" OR "congestive heart failure" OR HFrEF OR HFmrEF OR HFpEF) AND ("Metabolomics"[Mesh] OR metabolomic* OR metabolome* OR metabolom* OR metabonomic* OR "metabolic profiling") AND ("Mass Spectrometry"[Mesh] OR "mass spectrometry" OR "Nuclear Magnetic Resonance, Biomolecular"[Mesh] OR NMR OR "NMR spectroscopy" OR "Liquid Chromatography-Mass Spectrometry"[Mesh] OR "Gas Chromatography-Mass Spectrometry"[Mesh] OR "Gas Chromatography-mass spectrometry" OR "liquid chromatography" OR "gas chromatography" OR LC-MS" OR "LC-MS/MS" OR "GC-MS") AND ("2010/01/01"[dp] : "2024/12/31"[dp])	274
Cochrane	("Heart Failure" OR "cardiac failure" OR "myocardial failure" OR "congestive heart failure" OR HFrEF OR HFmrEF OR HFpEF) AND (Metabolomics OR metabolome OR "metabolic profiling") AND ("Mass Spectrometry" OR "Nuclear Magnetic Resonance, Biomolecular" OR " H-NMR" OR "NMR spectroscopy" OR "Liquid Chromatography-Mass Spectrometry" OR "Gas Chromatography-Mass Spectrometry" OR "liquid chromatography" OR "gas chromatography" OR "LC-MS" OR "LC-MS/MS" OR "GC-MS")	19
Scopus	TITLE-ABS-KEY ("Heart Failure" OR "cardiac failure" OR "myocardial failure" OR "congestive heart failure" OR HFrEF OR HFmrEF OR HFpEF) AND TITLE-ABS-KEY (Metabolomics OR metabolome OR metabolomic* OR metabolome* OR "metabolic profiling") AND TITLE-ABS-KEY ("Mass Spectrometry" OR "Nuclear Magnetic Resonance" OR NMR OR "NMR spectroscopy" OR "Liquid Chromatography-Mass Spectrometry" OR "Gas Chromatography-Mass Spectrometry" OR "liquid chromatography" OR "gas chromatography" OR "LC-MS" OR "LC-MS/MS" OR "GC-MS")	310
Web of Science	("Heart Failure" OR "cardiac failure" OR "myocardial failure" OR "congestive heart failure" OR HFrEF OR HFmrEF OR HFpEF) AND (Metabolomics OR metabolome OR "metabolic profiling") AND ("Mass Spectrometry" OR "Nuclear Magnetic Resonance, Biomolecular" OR NMR OR "NMR spectroscopy" OR "Liquid Chromatography-Mass Spectrometry" OR "Gas Chromatography-Mass Spectrometry" OR "liquid chromatography" OR "gas chromatography" OR "LC-MS" OR "LC-MS/MS" OR "GC-MS")	232

Table S2 Characteristics of included studies

Author, year, location	Study design	Sample size	Metabolomics domain(s)	Analytical methods	Findings	Comments
Tang et al, 2020, Taiwan[16]	Observational	HF (Event+)=31<br>HF (Event-)=30	Prognosis	Plasma, UT, UPLC-TOFMS	19 of the metabolites were significantly different between patients with and without event p<0.05	Metabolites showed better risk prediction than BNP (AUC 0.871 and 0.602 respectively)
Audurexiti et al, 2024, China[21]	Observational	HFpEF=30<br>Control=30	Mechanism	Plasma, UT, UHOLC-MS/MS	102 differentially expressed metabolites	Integrated proteomics and metabolomics analyses revealed distinct inflammatory and immune response pathways
de Oliveira et al, 2024, Brazil[22]	Observational	All=15 for HTx (8 Chagas disease, 7 IDCM), 12 donor heart	Mechanism	Plasma, GC-MS/MS	21 discriminating metabolites, (12 Chagas and 11 IDC) in the FFAs, aromatic amino acids (AAs), and components of the TCA cycle	The imbalance may explain the dissimilar clinical course of patients with Chagas disease
Yang et al, 2024, China[23]	Observational	HF=44,<br>Controls=30	Diagnostic	Serum, T, UPLC-Ms/MS, MRM	11 AAs differed between HF and controls, 6 based on HF severity, AUC>0.90 for 8 AAs Glutamic acid, Taurine, L-aspartic acid, L-ornithine, Ethanolamine, L-Serine, L-Sarcosine, and Cysteine	An objective diagnostic approach for early diagnosis and comprehension of the mechanisms of HF.
Selvaraj et al, 2024, multicenter, USA and Canada[24]	RCT	All =527<br>DEFINE-HF=234 and PRESERVE-HF	Diagnostic, therapeutic	Plasma, TF-MS	63 metabolites (45 acylcarnitines [ACs] 15 AAs, ketones, and no-esterified FAs). Two of the 12 PCA derived factors (ketone and SCACs/MCACs) increased with Dapagliflozin compared with placebo, Increase ACS was consistent across LVEF while Ketone decreased	Ketosis >500 um 4.5% Dapagliflozin vs 1.2% placebo (p=0.03), No treatment effects on AAs including BCAAs, Long-chain acylcarnitines (LCACs) increase and BCAAs decrease associated with worse outcomes, may serve as therapeutic targets across HF subtypes
Pouleur et al, 2024, Canada, Belgium[25]	RCT	HFrEF 221, HFpEF 136, controls 94, (BECAME-HF)	Diagnostic, prognostic	Serum and plasma, LC-M/MS	Myo-inositol was elevated in HFpEF>HFrEF (≥69.8µm HR 1.62 CI 1.05-2.5 for HFpEF)	Predicts poor clinical outcomes in HFpEF, SMT1 which transports myo-inositol represents a therapy target
Joo et al, 2024, US and South Korea[26]	Observational	All=1382<br>Disc=880,<br>Val. =502	Diagnostic	Plasma, ^{1}H-NMR	21 metabolites (lipoprotein subspecies, BCAAs, GlycA, Ketones, glucose, and citrate to derive metabolic risk score (MRS)	MRS was associated with an excess risk of death and improved risk stratification beyond established risk scores and clinical markers
Yang et al, 2024, China[27]	Observational	Angina=47<br>MI = 51<br>HF=80	Diagnostic, therapeutic	Plasma, UPLC-HRMC	97 endogenous metabolites,<br>28 diff angina and MI<br>32 between MI and HF	AUC Citrulline 0.62, citric acid 0.75, strearic acid 0.88, glycerophospholine 0.81, increased to 0.96 when combined, provide important targets for diagnosis and nutritional interventions
Zhou et al 2024, China[28]	Observational	Disc: HF=60, control=25, Val: HF=23, control=10)	Diagnostic	Serum, UT, UPLC-QTOF/MS	453 identified, 43 metabolites differed between HF and controls, the AAs pathway was most altered in HF.	Glcycodeoxycholate may serve as potential biomarker, with an AUC 0.99, Sensitivity 95%, Specificity 100% and may enhance early diagnosis of HF
Bekfani et al, 2022, Germany[29]	Observational	HFrEF=18<br>HFpEF=17<br>Control=20	Diagnostic	Serum, LC-MS/MS	Reduced AAs in HF vs controls except for aromatic AAs and BCAAs, elevated kynurenine (Kyn) and acylcarnitines (ACS) in HF vs controls	HFpEF with reduced muscle endurance (RME) had reduced ACs, HFrEF with RME had distorted AA metabolism (RME has different pathophysiology), Kyn may serve as marker of HF and RME (AUC 083, Sens 70%, Spec 83%)
Ma et al, 2022, China[30]	Observational	ADHF,<br>Disc=419<br>Val=386	Prognostic	Serum, UT, MS	27 Arachidonic acid metabolites, 14,15-DHET14/15-EET ratio strongest predictor of 1 year mortality<br>AA score (14,15-DHET/14,15-EET ratio, 14,15-DHET, PGD2 and 9-HETE were better AUC=0.85	Machine learning use showed incremental information beyond BNP. AA score reclassified 46.2% false-negative and 84.5% false-positive
Vignoli et al, 2022, Italy[31]	Observational	DCM=106 (80 alive, 26 died over 5 years	Prognostic	Serum, UT, ^{1}H-NMR	Metabolomic fingerprints discriminate patients who died (HR 5.71, p=0.00002) after adjustment	Metabolomics and LVEF (score) acts in synergy in predicting survival (HS 8.09, *p*=0000004), hence identifying HF patients at high risk of poor outcomes

Zhao et al, 2021, China, USA[32]	Observational	HF 628, HF=446 Non-HF=182	Prognostic	Dried blood, T, LC-MS/MS	102 metabolites (23 AAs, 35ACs, 44 ratios) 15 metabolites differed across the HF subgroups, Prognostic metabolic score (PMS) (developed from 9 metabolites) demonstrated better prognostic value (HR 1.62 vs NT-proBNP HR 1.23, p<0.001)	PMS 14exhibited superior prognostic value in predicting risk in HFmrEF and HFrEF, $p<0.001$ (AUC of PMS was superior to Log-NT-proBNP). May allow personalized treatment recommendation for each HF phenotype in future.
Wang et al, 2021, China and Germany[33]	Observational	HF=25 Control=25	Diagnostic	Serum, UT, HPLC LC-MS/MS	90 metabolites differed between HF and controls, serum and faecal metabolites correlated.	23 and 27 enriched metabolites may contribute to early screening in elderly patients with CHF
Oyetoro et al, 2024, UAS[34]	Observational	HF=1382	Diagnostic, prognostic	Plasma, UT, ^{1}H-NMR R	Higher ketone levels were observed in advanced HF HR 1.23 95% CI 1.05-1.44 between high and low group	Higher ketone body levels are associated with increased risk of death, especially in those with HFpEF
Ahmad et al, 2023, Australia[35]	Observational	HF=73 (EF<45%) (AHF=21, CHF=52) Controls=59	Diagnostic, therapeutic	Plasma UT, ^{1}H-NMR	HF has increased acetate, TMAO, sCD14, and CD163 compared with controls $p<0.05$	Distinct metabolomics and lipodomic profile in HF compared with controls at baseline
Belenkov et al, 2023, Russia[36]	Observational	HF = 79 Controls= 19 (CHD)	Prognostic	Pasma, T, FIA-MS/MS	30 ACs, 11 showing significant alterations. Positive correlations between MCACs and LCAC. Carnitine deficiency was found in patients with HF	ACs may be used to assess the severity of clinical manifestations and myocardial remodeling
Klobucar et al, 2023, Austria, Croatia[37]	Observational	HF=315 (AHF) Alive=197 Died=118)	Prognostic	Serum, UT, ^{1}H-NMR	8 of the 49 metabolites differed between those alive and those that died at 1 year	Low valine was independently associated with 1 year mortality (HR 0.73 95% CI 0.59-0.90; p=0.003). Valine >279.2µmol/L had higher survival AUC 0.65.
Park et al, 2023, USA[38]	Observational	TTR V1221 variant=57	Diagnosis	Plasma, UT, UHPLC-MS/MS	Identifies plasma metabolites associated with HF among TTR V1221 carriers	AUC 0.72 sensitivity =67, specificity =69, further studies to assess causal mechanisms in its pathogenesis
Hahn et al, 2023, USA[39]	Observational	HFpEF=38 (HTx donors), HFrEF=30 Non failing donor controls=20	Diagnostic	Plasma, T, HPLC Tr/quadriple MS	69 metabolites (AAs, organic acids, and ACs) did not differ between HF and controls. HFpEF combines both the metabolomics features of HF and obesity/DM	Despite more obesity and DM in HFpEF, values are same between HF and controls (but in the myocardium). Fuel inflexibility also occurs in HFpEF and is a target for therapy development
Gladding et al, 2022, New Zealand, Switzerland[40]	Observational	HFrEF=46 Controls=20 (NanoHF study)	Diagnostic (multi-omics)	Plasma, UT /T, LC-MS/GC-MS/SPME	28 metabolites by GCMS, 35 by LCMS and 4 by SPME.HF- several impaired pathways, commonly mitochondrial metabolism,	Metabolite panels (4 metabolites, arginine, tryptophan, Kyn, and acetone) are useful in diagnostic, monitoring of therapy, or nutritional interventions.
Zhang et al, 2022, China[41]	Observational	AF with HF=20 AF without HF=20	Diagnostic, prognostic (multi-omics)	Plasma, UT, LC-MS/MS	121 up-regulated and 14 down-regulated DEMs Multi-omics-glycolysis, gluconeogenesis, tyrosine metabolism and PPP were enriched	10 DEMs as biomarkers, AUC=0.94 9 DEMs distinguish between AF and HF and classification
Xu et al, 2022, China[42]	Observational	LVAD recipients=16 (improved=6, nICF=10)	Prognostic	Plasma, UT, UHPLC-MS/MS	1542 and 619 in positive and negative ion modes, 96 differed between nICF and ICF (baseline and LVAD support)	Identify differential features of nICF and ICF, screen potential predictive biomarkers of response to LVAD (tris(hydroxymethyl) aminomethane and 5-guanidino-3-methyl-2-oxopentanoic acid).
Anguita et al, 2022, Spain[43]	Observational	Acute HFpEF=8, stable HFpEF=8, Elderly controls=8, young controls=8	Diagnostic	Plasma, UT ^{1}H- NMR	Acute HFpEF: metabolic stress modifications (higher mitochondrial mass, ROS production, glycolysis): accumulation of lactate and formate, and reduced histidine	Low formate levels are markers of stable HFpEF Formate, lactate, and histidine differentiate the two HFpEF in the elderly, and may be used to monitor patients and suggest appropriate therapy
Truby et al, 2021, USA[44]	RCT (post-hoc)	All=664, DM=359, non-DM=305 (HF-ACTION)	Diagnostic, prognostic	Plasma,T, TFLC-MS/MS	45 ACs and 15 AAs, LCACs are associated with baseline exercise capacity and differ between those with and without DM	LCACs are associated with reduced exercise capacity and predict intermediate and hard outcomes (HR $0.80x10^{-8}$) even after adjustment (DM HR 0.64 $x10^{-9}$ vs 0.90 $x10^{-9}$, p=3.21)
Zhou et al, 2021, China[45]	Observational	HF=136 Stage A=49 (control), B=61, C+D=26	Diagnostic, prognostic	Plasma, UT, UHPLC-MS	142 lipids and 134 metabolomes were dysregulated in HF patients, (C and D, no difference, but between A and other stages. Some correlated with NT-proBNP and LVEF.	Complementary diagnostic indicators: 5 molecule panel, hypoxanthine, tryptophan, leucine, citric acid, and proline AUC0.8, potential as being prognostic

Xu et al, 2021, China[46]	Observational	AHF=89<br>Control=80	Diagnostic	Plasma, UT, UPHLC-MS	AHF metabolites differed from controls (AAs, FAs, lipids, nucleosides, and derivatives)	Adenine, glutamic acid, pseudouridine, y-glutaminecysteine (AUC 0.995, 0.932,0.920, and 0.900), similar to BNP AUC 0.978
Zordoky et al, 2015, Canada[47]	Observational	HFpEF=24<br>HFrEF=24<br>Non-HF controls=38<br>(Alberta HEART)	Diagnostic	Serum, LC-MS/MS and ^{1}H-NMR	HFpEF higher ACs, carnitine, betaine, AAs, and lower levels of phosphatidylcholine and lysophospo, sphingomyelins.<br>MCACs and LCACs differed between HFpEF and HFrEF	HFpEF differed from HFrEF metabolically, and the 2 panels (AUC0.942 and 0.981) can separate HFpEF from non-HF controls and HFrEF
Mueller-Hennessen et al, 2017, Germany[48]	Observational	HF=22<br>Controls=19	Diagnostic, prognostic	Plasma, UT, GC-MS and LC-MS/MS	252 metabolites, 164 of them showed profound abnormal lipids, AAs metabolism and a few exercise specific changes	Metabolites could differentiate male HF patients due to IICM from controls and improved predictive power when combined with LVEF and NT-proBNP
Marcinkiewicz-Siemion et al, 2020, Poland[49]	Observational	Chronic HFrEF=67<br>Controls=39	Diagnostic	Serum, UT, LC-QTOF-MS	8 metabolites showed predictive value in HFrEF, Metabolite panel AUC compares to BNP 0.85 vs 0.82	UT and machine learning aid HFrEF diagnostic workup. Metabolites provided comparable BNP diagnostic value
Kretzschmar et al, 2024, Germany[50]	Observational	ADHF=15<br>CHF=50<br>Controls=13	Diagnostic, prognostic	Plasma, UT, UPLC-MS/MS	630 metabolites, 57 (12%) were altered in ADHF, 22 in CHF, 1-MetHis and 3-IPA consistently altered in both ADHF and CHF AUC ≥0.8	Symmetrical DMA was a suitable marker for ADHF and Kyn for CHF AUC ≥0.85
Hundertmark et al, 2023, Germany, UK[51]	RCT	HFrEF=17, control=19<br>HFpEF=18, Control-18<br>(EMPA-VISION)	Mechanistic, Therapeutic	Serum, T, MS	19 metabolites], and no change in the metabolomes after 12 weeks of empagliflozin	Findings did not confirm the thrifty fuel hypothesis with SGLT2i in HF
Hannemann et al, 2024, Germany[52]	Observational	Disc= DCM =297 M, 71 W<br>Val. cohort= 93 M	Diagnostic	Plasma, UT, LC-MS/MS	163 metabolites and proteins, and integrated analysis showed close relations between proteins and metabolites in lipid metabolism, Kyn most important, dependent on sex	Integration may help gain insights into the alterations associated with DCM
Luan et al, 2013, China[53]	Observational	HF= 23, CAD= 23<br>Controls= 23	Mechanism, diagnostic	Serum, UT, HPLC-MS	FAs, sphingolipids, and AAs derivatives were disturbed in HF patients compared with CAD	Lipid molecules associated with energy metabolism may play key roles in the development of HF
Liu et al, 2021, China[54]	Observational	HF=96 (CHD=67, DCM=13, VHD=16)<br>Controls=97	Diagnostic, prognostic	Dried blood spot, T, LC-MS/MS	23 AAs and 26 carnitines assessed, 9 metabolites relate to HF, Glutamine and hydroxyhexadecanoyl-carnitine (HDC) varied between CHD and DCM and HDC and aspartic acid between CHD and VHD	AAs and LCACs are associated with HF progression and may help differentiate CHD, DCM, and VHD in early stages<br>May provide new diagnostic and therapeutic targets
Wegermann et al, 2023, USA[55]	Observational	HFpEF=37, no=52<br>HFpEF, no fatigue yes=30, no=59<br>DD yes=47, no=42	Diagnostic, Targets for therapy	Serum, UT, UHPLC-MS/MS	1151 metabolites detected, 656 were finally analyzed (8 pathways), and 53 were increased in HFpEF, mostly lipids ($p<0.05$), but AAs (2 cysteine metabolites) and carbohydrates were low.	Abnormalities of the lipid pathway might be the link between biopsy-proven NAFLD and HFpEF<br>Might help development of biomarkers and treatments targets
Petruhnova et al, 2024, Russia[56]	Observational	HFpEF=82, HHD and CHD=45, controls=27	Diagnostic, prognostic	Plasma, T, UPLC-MS	84 AAs, reduced threonine in HFpEF compared with others (inversely correlated with glucose; Diabetes vs no DM $p=0.029$)	Threonine should be considered for diagnostic and prognostic patients with HFpEF
Hilse et al, 2021, Germany[57]	Observational	DCM=20<br>ICM=20, controls=20	Diagnostic, prognostic	Plasma, LC-MS/MS	188 metabolites were measured, of which 63 were altered and three returned to pre-LVAD concentrations. AAs and biogenic amines differed between DCM and ICM	There is reversal of metabolite abnormalities after LVAD implantation, showed the disease pattern of ICM and DCM, diagnostic and prognostic value
Peng et al, 2019, China[58]	Observational	AHF=411<br>High risk group=206<br>Low risk group=205	Prognostic, monitoring	Plasma, T, HILIC-LC/MS/MS	High baseline 2-oxoglutarate (2OG) was associated with short-term hospitalization and all-cause mortality independent of NT-proBNP and eGFR	2OG may be helpful for risk stratification and treatment monitoring in AHF
Selvaraj et al, 2023, Canada, USA[59]	RCT	DFINE-HF=234	Mechanism, prognostic	Plasma, T, LC-MS/MS	63 metabolites measured at baseline and 12 weeks (45 ACs, 15 AAs, and 3 other metabolites. SGLT2i decreases SCACs/MCACs and KB vs placebo,	SGLT2i causes alteration in KB and fatty acid biology, LCACs, decarboxylated LCACs, and aromatic AAs are associated with adverse HF events and higher NT-proBNP
Wang et al, 2013, China[60]	Observational	CHF=39<br>Controls=15	Diagnostic, mechanistic	Plasma, UT, ^{1}H-NMR	Demonstrated hyperlipidaemia, altered energy metabolism, and others occur in CHF	Use to identify diagnostic biomarker and insight into CHF metabolic processes

Du et al, 2014, China[61]	Observational	HF=46<br>Controls=15	Diagnostic, mechanistic	Serum, UT, ^{1}H-NMR	Significant differences in metabolic groups of HF patients with different metabolic energy equivalent (MEE) especially 3-hydroxybutyrate, acetone, and succinate	Use as potential biomarkers of myocardial energy metabolism in HF patients.
Ahmad et al, 2017, USA[62]	RCT (post-hoc)	HF-ACTION<br>End-stage HF=41 (LVAD)<br>CHF=452 of 2331(controls)	Prognostic, therapeutic	Plasma, T, LC-MS/MS	45 ACs and 15 AAs, LCACs (C16, C18:1, and C18:2 and CAs metabolites were higher in patients with end-stage HF before LVAD placement and decreased at 90 days	LCACs are independently associated with clinical outcomes (cardiopulmonary fitness, hospitalization, death etc.) and decreased after LVAD therapy
Alexander et al, 2010, USA[63]	Observational	Primary DCM=39<br>Controls=31	Diagnostic, prognosis	Plasma, T, GC/MS, UHPLC/MS	61 metabolites, decreased sex steroids, glutamine, threonine, and histidine, increased TCA cycle, and FAs oxidation metabolites.	Use for early disease detection, prognosis, and treatment of HF patients
Katano et al, 2022, Japan[64]	Observational	HF and DM=81<br>SGLT2i=29, no=52	Mechanism	Plasma, T, UPLC-MS	Elevated essential AAs, leucine, and histidine and non-essential AAs, β-aminoisobutyric acid (BAIBA)in patients taking SGLT2i	Insight into the mechanism of SGLT2i cardioprotection
Kouzu et al, 2021, Japan[65]	Observational	HF=301<br>Event (yes)=40<br>Event (no)=261	Prognostic	Plasma, T, UPLC-MS	34 metabolites including 10 essential AAs<br>3-mthylhistidine (3-Me-His), β-alanine, valine, tryptophan, and hydroxyproline were associated with adverse events in HF, (3-Me-His, β-alanine, and valine were independently associated after adjustment)	Improved predictive ability for adverse events when 3-Me-His is combined with either valine or β-alanine. Also suggest a role for wasting and malnutrition in poor prognosis
Naeem et al, 2024, USA[66]	Observational	HFpEF=357<br>HFrEF=219<br>Controls=211	Diagnostic, therapeutic	Plasma, T, UHPLC-quadrupole MS	90 metabolites, (54 ACs, 28 AAs, 8 organic acids)<br>MCACs, LCACs, and 3-hydoxybutyrate increased in HFrEF than HFpEF and controls and elevated metabolites of BCAAs e.g., ADMA in HFpEF and SDMA in all	NT-proBNP correlated with 3HBA and C4-OH across HF groups. Help with diagnosis and phenotype-specific therapeutic and responses by the HF groups
Chen et al, 2023, China[67]	Observational	IHD-HF=63<br>DCM=98<br>Control=48	Diagnostic, prognostic	Plasma, T, UHPLC-MS/MS	All 25 ACs were higher in HF than controls, 20 independently associated with HF diagnosis, and about 7 increased the probability of DCM diagnosis<br>AUC increased from 0.77 to 0.83 when isobutyl-L-carnitine and stearoyl-L-carnitine were added to conventional factors	C18:1 and C18:2 predict all-cause mortality whereas C18:2 and C4DC predict rehospitalization.<br>Serum carnitines could serve as diagnostic, prognostic, and help identify the aetiology of HF
Conners et al, 2023, USA[68]	Observational	All=1382, MVX1=171, MVX2=339, VX3N=445, MVXN=4427	Diagnostic, prognostic	Plasma, T, ^{1}H-NMR	Metabolic vulnerability index (MVX) made up of GlycA, small HDL, and a composite measure of inflammation and metabolic malnutrition	MVX is associated with increased mortality; patients with the highest MVX are 3x more likely to die after adjustment for MAAGGIC and other biomarkers
Teis et al, 2021, China[69]	Observational	All=422, CV death=120, Non- CV death=80, Survivors=222	Prognosis	Serum, T, ^{1}H-NMR	Significant derangements in mean HDL-Sz and HDL-C/P ratio among CV death compared to survivors after adjustment	Help in refining HF risk stratification,
Hen et al, 2021, China[70]	Observational	All=699, HF A=95, HF B=116, HF C383, Control=75	Mechanism	Plasma, T, LC-MS/MS	Elevated levels of phenylalanine (Phe) and tyrosine but a decreased ratio of tyrosine to Phe from early to late stages of HF.	Values of Phe, tyrosine, Met-SO, total bilirubin, and CRP decreased after LVEF recovered and were mainly related to CRP, suggestion that inflammation played a role in Phe increase
Chen et al, 2020, Taiwan[71]	Observational	HF stage C=79 (improved=42, not improved=37), Controls=51	Prognostic	Plasma, UT, LC-MC	HF had higher levels of ACs (40) of all chain length.<br>FA metabolism improved in those whose cardiac function improved on follow-up	Changes in SCACs profiles are independently associated with improvement in cardiac systolic function.
Nayak et al, 2020, USA[72]	Observational	ICM=154<br>Non-ICM=320 (control)	Prognostic	Plasma, T, LC-MS	N8AS > ICM than CAD without ICM and NICM, N8AS is associated with higher mortality in ICM, independent of BNP, and is associated with a greater risk of incident HF	Higher levels are predictive of mortality and HF hospitalizations in patients with ICM, and with incident HF in those without HF

Zhao et al, 2020, China[73]	Observational	HF=117<br>Non-HF=118	Diagnosis, prognosis	Dried blood spot, UT, direct infusion MS	49 metabolites (23 AAs and 29 ACs), 8 (5 increased, 3 decreased) differed between HF and non-HF, 7 with a sensitivity and specificity (0.8974 and 8475)	Alternative screening method to predict prognosis
Guo et al, 2020, China[74]	Observational	HF=143 Controls=34<br>Val. cohort=74	Diagnostic	Serum, UT, GC-MS	5 metabolites were high in NYHA II-IV, 11 in NYHA 1. Glutamine and tyrosine help distinguish NYHA and those with normal NT-proBNP.	Glutamine showed the highest diagnostic potential, which was demonstrated in the validation cohort.
Hayashi et al, 2018, Japan[75]	Observational	Decomp HF=22<br>Comp HF=22,<br>Controls=11	Diagnostic	Plasma,<br>CE-TOFMS	TMAO in decompensated HF was higher than control, p=0.003 and Comp HF vs controls p=0.004<br>Positive correlation between Escherichia/Shigella and TMAO and IS levels (more in Decomp than Comp HF) p<0.05 for both	Gut microbiome and microbiome related metabolites are altered in patients with HF, and positively correlated with TMAO and indole sulphate (IS).
Wang et al, 2018, Taiwan[76]	Observational	Stage A=93, B=120, C=138, and D=101, Controls=129	Diagnosis, prognosis	Plasma, UT, UPLC-MS/MS, FIA-MS/MS	21 metabolites assessed, Histidine, ornithine, and phenylalanine (HOP) correlated with NT-proBNP and better discrimination of patients at different stages	HOP panel provides an additive diagnostic and prognostic value to NT-proBNP and traditional risk factors
Du et al, 2018, China[77]	Observational	STEMI without event=85<br>STEMI with event=53	Diagnosis, prognostic	Plasma, UT, UHPLC-MS/MS	26 metabolites were assessed, but 5 (BCAAs), tyrosine and phenylalanine were independent predictors of death and HHF after adjusting for risk factors, AUC=0.80 > NT-proBNP=0.72,	BCAAs are associated with long-term adverse outcomes and could be a novel pathophysiological mechanism in clinical outcomes after myocardial infarction
Marcinkiewicz et al, 2018, Poland[78]	Observational	Der. (HF=36, controls=19)<br>Val. (HF=31, controls=20)	Diagnosis, mechanism	Serum, UT, LC-QTOF-MS	110 and 60 dysregulated metabolites (lipid, FAs, amines, uric acid, and AAs in the derivation and validation cohort. Phospholipid deficits correlate with increased ACs, age, renal function, uric acid, and cholesterol.	Suggest HF metabolic changes may play a role in HF pathogenesis.
Lanfear et al, 2018, etherlands, USA[79]	Observational	Der. HFrEF=516<br>Val. HFrEF=516	Diagnostic, prognostic	Plasma, T, HPLC-MS, GC/MS	23 AAs, 8 organic acids (OAs), and ACs were analyzed and showed strong phenotypic associations and varied with severity	The PMP (13 assessed metabolites) is a strong predictor of survival in HF even after accounting for NT-proBNP and clinical risk factors
Ruiz et al, 2017, Canada, France, and USA[80]	Observational	Der. (HF=68), controls=72<br>Val.= HF=8, controls=8	Mechanism	Plasma, T, LC-MS/MS	Altered ACs (53) irrespective of chain length especially in HF patients, mitochondria and peroxisomes.	Highlight a novel mechanism contributing to global lipid perturbations in HF.
Hunter et al, 2016, USA[81]	Observational	HFrEF=279, HFpEF=282, no-HF (controls)=191 (parts of CATHGEN)	Diagnostic	Plasma, T, LC-MS/MS	63 metabolites were analyzed and reduced to 14 by PCA elevated in HFpEF and HFrEF compared to no-HF controls. LCACs are linearly higher in HFpEF than HFrEF	Elucidate the specific pathway in HF and suggest a shared metabolomics mechanism along the LVEF spectrum
Deidda et al, 2015, Italy[82]	Observational	HF (EF<35%) =15<br>HF (EF35-50%) =9<br>Controls=9	Diagnostic	Plasma, UT,<br>^{1}H-NMR	Identified a metabolic fingerprint specific to each HF stage independent of BNP	Metabolic screening in association with traditional cardiac parameters valuable in evaluating the pathophysiology of HF and ascertaining alterations associated with HF
Cheng et al, 2015, Taiwan[83]	Observational	Disc. Stage B=67, C=73, Control=51<br>Val. Stage C=218, Control=63	Diagnostic, prognostic	Plasma, UT, UPLC-MS	4 metabolites histidine, phenylalanine, spermidine, and phosphatidylcholine C34:4 differentiate stage c from controls, similar to BNP, AUC 0.99<br>Another set of 4 metabolites had optimal prognostic value, better than BNP	HF is associated with abnormalities in multiple metabolic pathways, which aids in understanding the pathogenesis and prognostic value independent of BNP
Padeletti et al, 2015, Italy[84]	Observational	HF=32, Controls=39	Diagnostic, prognostic	Serum, UT,<br>^{1}- NMR	Metabolomes differed between HF and controls but could not differentiate ICM from NICM	Metabolomics profiling cannot identify CRT responders.
Tenori et al, 2013, Italy[85]	Observational	HF=185<br>Controls=111	Diagnostic	Serum,<br>^{1}H NMR	HF patients can be characterized by metabolomes compared to healthy controls.	Greater than 86% accuracy to discriminate HF from healthy controls
Desmoulin et al, 2013, France[86]	Observational	HF=126, No event=98<br>Event (30 days) =28	prognostic	Plasma, T,<br>^{1}H-NMR	HF patients exhibit high plasma lactate and low total cholesterol (Chol) concentration and a high Lac/Chol ratio.	Plasma Lac/Chol ratio is a simple and objective short-term prognostic value.

Wang et al, 2016, Taiwan[87]	Observational	HF=136<br>Controls=51	Prognostic	Plasma, UPHLC-MS/MS	p-cresyl sulfate (PCS), indoxyl sulfate, and arginine are associated with a high rate of composite events especially if ≥50 µm. PCS was an independent predictor (HR 1.06, *p*=0.02).	PCS provided the best prognostic factor among the few AAs derived, metabolites including dimethyl arginine/arginine ratio are independent of BNP.
Steffens et al 2010, USA[88]	RCT	HF with depression=40<br>HF no depression=40	Diagnostic	Plasma, UT, LC-GC/MS	Metabolomes identified 205 known and 218 unknown metabolites and differed (elevated) between the two groups, but have reduced KB but increased dicarboxylic acid formation	Neurotransmitter and FA metabolism are associated with the depressed state, but the role in HF is unclear
Wang et al, 2017, USA[89]	RCT	HFpEF=113<br>Controls=103<br>(RELAX sub-study)	Diagnostic, prognostic	Plasma, T, LC-TOD-MS/MS	Profiled 45 ACs, 15 AAs, and 5 conventional metabolites, 7 metabolites differed between groups (decreased alanine and proline, increased SCACs and LCACs), associated with Endothelin-1 and renal function and worse clinical score at 24 weeks	Treatment with Sildenafil significantly increased LCACs and SCACs metabolite paralleling worsening collagen metabolism and an increase in adverse clinical markers. Provide candidates for biomarkers associated with heart failure
Jovanovic et al, 2024, Germany[90]	Observational	HFpEF=18<br>Controls=12	Diagnostic	Plasma, LC-MS/MS	No significant differences between HFpEF and controls concerning lipid species or lipids binned by carbon chain length or number of bonds	Could not detect specific lipids as biomarkers of HFpEF, Demonstrated link between lipidomic parameters and some of the symptoms and covariates of HFpEF
Tang et al, 2017, Taiwan[91]	Observational	Disc. HF Stage A=29, B=29, C=29, Control=28<br>Val HFpEF=87, Controls=28	Mechanism	Erythrocyte, LC-MS/MS	Erythrocyte lipid of HFpEF differed from controls, notably 7-ketocholesterol (7KCh).<br>7KCh promotes the reactive oxygen species pathway	7KCh could be a risk factor for HF and hence may be implicated in the pathophysiology

Abbreviations: AAs:= amino acids, AAAs:= aromatic amino acids, Acs:= acylcarnitines, AUC:= area under the curve, BCAAs:= branch-chain amino acids, CV=cardiovascular, DEMs= , Der:= derivation cohort, HF:= heart failure, HHF:= hospitalization for heart failure, HFmrEF:= heart failure with mildly reduced ejection fraction, HFpEF:= heart failure with preserved ejection fraction, HFrEF:= heart failure with reduced ejection fraction, ICM:= ischaemic cardiomyopathy, KB:= ketone bodies, LCACs:= long chain acylcarnitines, LC-MS/MS:= liquid chromatography tandem mass spectroscopy, MCACs:= medium chain acylcarnitines, ^{1}H-NMR:= nuclear magnetic resonance nICF:= non-improved ischaemic heart failure, NICM:= nonischaemic cardiomyopathy, PCA:= principal components analysis, Phe:= phenylalanine, RCT:= randomised controlled trial, SCACs:= short chain acylcarnitines, T:= targeted, TMAO:= Trimethylamine N-oxide, UT:= untargeted, Val:= validation cohort
